# Vascular Endothelial Injury and Apoptosis in Rats with Severe Acute Pancreatitis

**DOI:** 10.1155/2015/235017

**Published:** 2015-01-22

**Authors:** Ning Ge, Qing Xia, Zhong-Hua Yang, Qun-Fang Ding, Zhi Zeng

**Affiliations:** ^1^Division of Geriatric Medicine, West China Hospital of Sichuan University, Chengdu, Sichuan 610041, China; ^2^Chinese Medicine Department, West China Hospital of Sichuan University, Chengdu, Sichuan 610041, China; ^3^Cardiac Medicine Department, West China Hospital of Sichuan University, Chengdu, Sichuan 610041, China

## Abstract

We explored mechanisms of vascular endothelial injury that lead to systemic multiple organ failure by detecting the soluble endothelial protein C receptor (sEPCR), von Willebrand factor (vWF), serum nitric oxide (NO), and tumor necrosis factor alpha (TNF-*α*) and Bcl-2 mRNA and Bax mRNA expression in a severe acute pancreatitis (SAP) rat model. Compared to controls, the levels of TNF-*α*, vWF, and sEPCR were significantly increased in the experimental group at 12 hours and 24 hours and the NO level was significantly decreased. After 12 hours, the aortic endothelial apoptosis index and Bax mRNA expression in aortic endothelial cells had increased in the experimental group, but Bcl-2 mRNA levels had decreased. All these changes appeared at both 12 h and 24 hours. The results indicated that vascular endothelial injury and apoptosis markers were elevated in SAP. Endothelial injury and increased apoptosis in the experimental group were related to the increased expression of TNF-*α*.

## 1. Introduction

Severe acute pancreatitis (SAP) progresses rapidly, often induces systemic multiple organ failure, and has a mortality rate between 10 and 30%. Scientists originally thought that increased trypsin activity triggered acute pancreatitis and hypothesized that SAP developed when pancreatic enzymes were released into the circulatory system [1]. Because trypsin inhibitors did not successfully prevent or reverse the effects of SAP [2], further research was performed and the progression of acute pancreatitis to SAP has recently been linked to pancreatic microvascular spasm, changes in hemodynamics, and the excessive activation of inflammatory mediators [2], possibly by hemorrhagic changes [3–6].

The specific mechanisms of circulation disturbance in SAP remain unclear. In order to delineate these mechanisms, we measured the amount of pathological injury to the pancreas and the levels of tumor necrosis factor-*α* (TNF-*α*), von Willebrand factor (vWF), soluble endothelial protein C receptor (sEPCR), serum nitric oxide (NO), and Bcl-2 mRNA and Bax mRNA in the blood of a rat model of SAP.

## 2. Materials and Methods

The study protocol was approved by the Animal Care Committee of West China Hospital of Sichuan University. A total of 48 two- to three-month-old Sprague-Dawley (S-D) rats that weighed between 180 and 200 g were randomly divided into a control group and an experimental group. After surgery, 12 rats in each group were sacrificed at 12 hours and 12 were sacrificed at 24 hours, making four groups of 12 animals each.

The rats were housed six to a cage at room temperature on a 12 : 12 light/dark cycle with free access to food and water for five days before the study. Food, but not water, was withdrawn 12 hours before the study started.

The rats were anesthetized with 2% ketamine (80 mg/kg) injected into the intraperitoneal cavity. SAP was produced in the experimental group by two injections of an 8% solution of L-arginine (pH 7.0, Sigma Co., USA) (4.4 mg/g of body weight, administered intraperitoneal cavity) given one hour apart [7, 8]. The control group received two injections of an equal amount of 0.9% normal saline.

Twelve rats from each group were sacrificed under anesthesia 12 h and 24 h after the injections. Blood samples were taken and centrifuged for 10 min at 3,000 rpm. The serum was preserved at −80°C. The aorta was removed and preserved at −70°C after being frozen in liquid nitrogen for PCR analysis. Part of the aorta was fixed with 4% formalin and embedded in paraffin sections for a TUNEL test.

Tissue extracted from the head of the pancreas was fixed with a 40 g/L formalin solution, paraffin-embedded, sliced, and stained with hematoxylin and eosin (HE). A pathologist examined the tissue and gave it a pathology score according to the Kusske standard [9] based on edema, cell inflammation, and necrosis. ELISA was used to measure sEPCR, vWF, NO, and TNF-*α*. A reverse transcriptase polymerase chain reaction study (RT-PCR) was performed according to the manufacturer's instructions. Aortic endothelial apoptosis was detected with a TA200* In Situ* Apoptosis Detection Kit (R&D Co., USA).

### 2.1. Statistical Analysis

Results from* in vitro* studies are expressed as mean ± SD. After a homogeneity test of variance, a comparison between the means of two samples was made using two *t*-tests of group design.

## 3. Results

**Figure 1:**
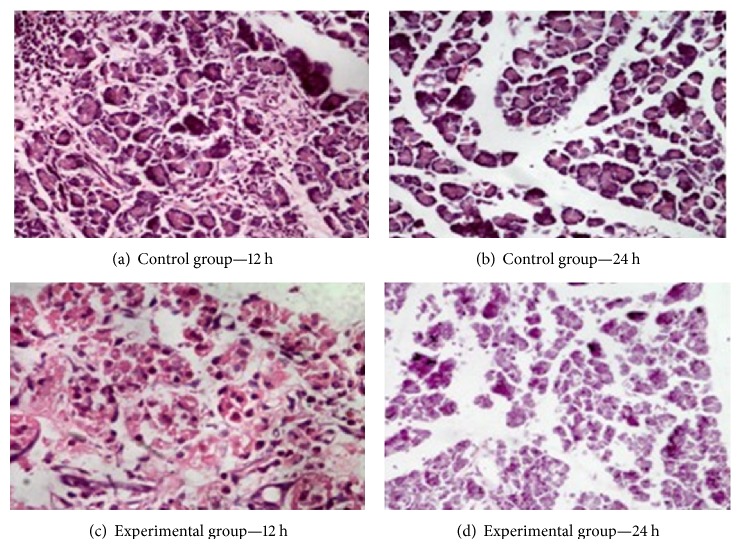
Pancreatic tissue HE staining.

Pancreatic tissue from the control group revealed no edema or inflammatory cell infiltration at any time point (Table 1). In the experimental group, there were partial pancreatic tissue necrosis and gland structure disappearance at 12 h, along with red-stained, nonstructural material formation and a large amount of inflammatory cell infiltration and bleeding. The glands that were not necrotic were edematous. After 24 hours, the pancreatic acinar structure of the rats in the experimental group had disappeared and congestive edema was visible between the cells and the acinar lobules. A small amount of inflammatory cell infiltration was occasionally observed (Figure 1).

After 12 h, the levels of vWF, sEPCR, and TNF-*α* were significantly increased in the experimental group compared to the control group (1.12 ± 0.17 versus 0.531 ± 0.22, *P* < 0.05; 3.749 ± 0.27 versus 2.254 ± 0.175, *P* < 0.05; and 70.452 ± 2.927 versus 25.28 ± 1.420, *P* < 0.05, resp.) (Table 2). These levels were the same at 24 h. The NO level in the experimental group was significantly lower than the NO level in the control group at 12 h (2.729 ± 0.613 versus 5.121 ± 1.562, *P* < 0.05), and this discrepancy was also observed at 24 h (Table 2).

The pancreatic pathological score in the experimental group was significantly increased compared to the control group at 12 h (6.77 ± 1.37 versus 3.67 ± 0.817, *P* < 0.05) and remained at this level at 24 h (Table 2).

**Figure 2:**
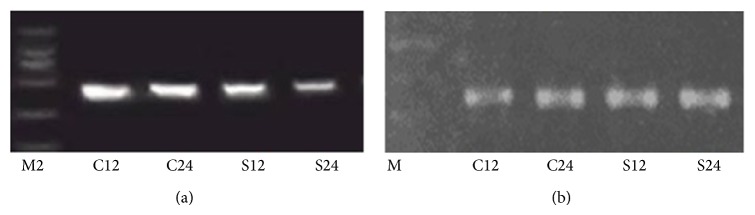
(a) BCL-2 and (b) Bax RT-PCR analysis of the aortic endothelium in the experimental and control groups after 12 hours and 24 hours.

**Figure 3:**
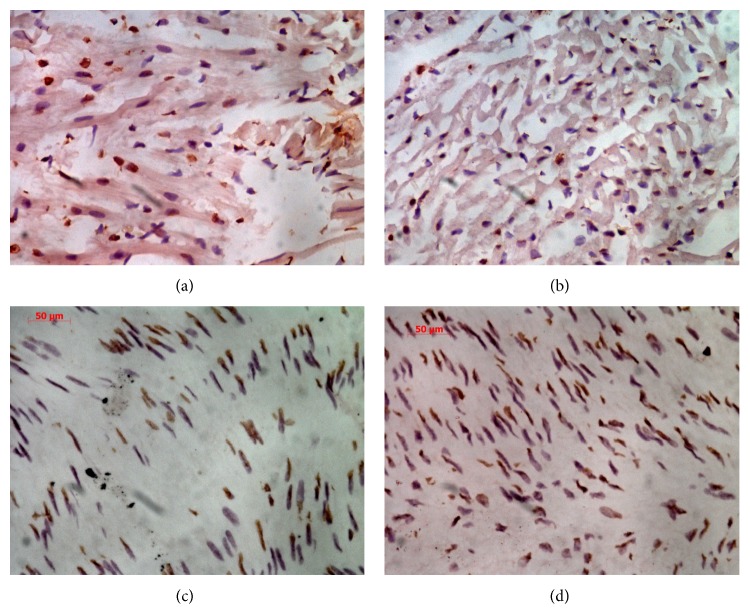
Aortic endothelial cell apoptosis TUNEL analysis results. (a), (b) Aortic endothelial cell apoptosis in the control group after 12 h (a) and 24 h (b). (c), (d) Aortic endothelial cell apoptosis in the experimental group after 12 h (c) and 24 h (d).

There were only a few apoptotic cells in the aortic endothelia of the control group at 12 h, but the number of these cells in the experimental group was significantly higher at 12 h and remained higher at 24 h (51.47 ± 23.56 versus 5.87 ± 0.15, *P* < 0.01) (Figure 3, Table 2). The experimental group had significantly less Bcl-2 mRNA expression (0.00224 ± 0.00049 versus 0.00985 ± 0.0039, *P* < 0.05) and significantly more Bax mRNA expression (0.00897 ± 0.0036 versus 0.00184 ± 0.00051, *P* < 0.05) than the control group at 12 h, and this was also true at 24 h. The Bcl-2/Bax mRNA ratio was lower in the experimental group than the control group (*P* < 0.05) at both 12 and 24 h (Figure 2).

## 4. Discussion

Despite a great deal of basic science research, the exact mechanism of SAP remains unknown. Treatment is mostly ineffective, and the SAP mortality rate remains high at 10–30%. Many researchers attribute multiorgan failure in SAP to a combination of primary pancreatic enzyme activation and pancreatic autodigestion, followed by secondary immune-mediated damage and the breakdown of vascular barriers. Vascular endothelial cell injury and functional changes may be the core mechanism of circulatory disturbances in SAP; but whether vascular endothelial injury actually exists in SAP and whether this injury triggers multiorgan failure have not been extensively researched.

We chose a classic rat SAP model to observe changes in the serological markers of arterial endothelial injury and endothelial apoptosis. We also explored whether the mechanism of vascular endothelial injury and apoptosis in SAP is associated with increased TNF-*α* expression.

Rising sEPCR and vWF concentrations in plasma are a sign of vascular endothelial cell injury. vWF has strong sensitivity but lacks specificity because levels fluctuate with mild stimulation [10, 11]. sEPCR maintains a stable concentration, does not change with age, and is not affected by mild stimulation [12–15]. Together, these two markers can accurately measure endothelial cell trauma.

The vWF and sEPCR levels in our experimental group were significantly higher than the levels in the control group at both 12 h and 24 h (*P* < 0.05). This suggests that SAP injured the vascular endothelial cells and the degree of injury rose as the condition progressed. We think that both vWF and sEPCR can be used to determine the severity of SAP and formulate a probable prognosis.

Causes of endothelial cell injury in SAP are complicated and may be associated with a number of factors. One factor is oxidative stress. Activated endothelial cells produce large amounts of reactive oxygen species that cause oxidative damage and dysfunction in the endothelium unless they are scavenged and removed from the system [16]. SAP interferes with this scavenging and removal. Activated endothelial cells also produce select adhesion molecules that release cytokines that damage the endothelium [17–20]. Endothelin (ET) is produced in massive quantities in acute pancreatitis and increases as the disease progresses. When the ratio of ET to NO is not balanced, peripheral resistance in the circulatory system increases and elevates the blood pressure. Elevated blood pressure aggravates endothelial cell injury [21]. The pancreas releases many enzymes during SAP that accelerate pancreatic gland necrosis and travel through the circulation to damage vascular endothelial cells as well.

In recent years, research has focused on the relationship between cell factors, such as tumor necrosis factor-*α* (TNF-*α*) and SAP. TNF-*α* is a cytokine produced by mononuclear macrophages that has a very wide range of biological activity. TNF receptors exist on the vascular endothelial surface and research according to [22] suggests that TNF-*α* has a direct cytotoxic effect on vascular endothelial cells [23].

At 12 h, the serum TNF-*α* concentration in our experimental group was significantly higher than that of the control group (*P* < 0.05), and as the TNF-*α* concentration rose so did the rats' vascular endothelial injury factor. Both concentrations continued to rise and reached their highest levels at 24 h.

We concluded that TNF-*α* played an important role in arterial endothelial injury in our SAP rat model. In this study, we were not sure about the causality between the tumor necrosis factor and vascular endothelial injury; previous researches have confirmed the tumor necrosis factor through the four aspects of the mechanism damage of vascular endothelium. Four factors may contribute to the mechanism. First, TNF-*α* can cause direct damage [24]. Second, TNF-*α* can activate the lysosomal enzyme complex and increase cell surface protease expression, which further damages endothelial cells [25, 26]. Third, TNF-*α* can significantly promote platelet growth and release. A high platelet count signals injury in the body, and this signal attracts monocytes that transform into macrophages to clean up cellular debris. Large amounts of TNF-*α* are released in the process, creating more injury that attracts more monocytes and damages the vascular endothelium in a repetitive loop. Fourth, the increased expression of TNF-*α* increases the permeability of vascular endothelial cells, resulting in vascular endothelial dysfunction [27, 28].

Bcl-2 family proteins are the regulators of apoptosis; this family of interacting partners includes inhibitors and inducers of cell death. Together they regulate and mediate the process by which mitochondria contribute to cell death known as the intrinsic apoptosis pathway [29]. Bcl-2 and Bax are important apoptosis regulatory genes of Bcl-2 family. The overexpression of Bcl-2 inhibits apoptosis and the overexpression of Bax accelerates apoptosis [30–33]. In our study, aortic endothelial cell apoptosis was significantly increased in the experimental group after 12 hours and continued to increase up to 24 hours. Controls had only small amounts of aortic endothelial cell apoptosis and there was a significant difference between the apoptosis indexes of the two groups (*P* < 0.05). Bcl-2 mRNA expression had decreased significantly and Bax mRNA expression had increased significantly 24 h after SAP was induced. The Bcl-2/Bax mRNA ratio was lower in the experimental group than in the control group at 12 h and continued to drop until 24 h (*P* < 0.05).

These results suggest that aortic endothelial cell apoptosis in rats with SAP is associated with alterations in the Bcl-2/Bax mRNA expression ratio, which is supported by other researches [33]. Endothelial cell apoptosis in SAP rats was related to increased serum TNF-*α*. In our study, we did not determine the aetiological relation between endothelial cell apoptosis and TNF-*α*; previous researches demonstrated that TNF-*α* induces endothelial cell apoptosis by calcium overload, lipid peroxidation, and the function of the redox imbalance [34–36]. In addition, a large number of cytotoxic-free oxygen radicals were produced that caused endothelial damage and cell apoptosis [37].

## 5. Conclusions

Vascular endothelial injury markers and Bax markers of vascular endothelial apoptosis increased in SAP, but Bcl-2 markers of vascular endothelial apoptosis decreased. This indicates that multiple organ failure in SAP is associated with direct damage to the pancreas as well as circulatory disturbances, and endothelial injury and increased apoptosis are associated with the increased expression of TNF-*α*.

## Figures and Tables

**Table 1:** Pathological score standard.

Index	Classification standards	Score
Edema	Interlobular local edema	1
	Interlobular diffuse edema	2
	Gland disorder, separation	3

Inflammatory cells	No	0

Infiltration	No	0
	Confined to the catheter	1
	Limited to the parenchyma (<50%)	2
	Limited to the parenchyma (>50%)	3

Acinar necrosis	No	0
	Necrosis around the catheter (<5%)	1
	Focal necrosis (5%~20%)	2
	Substantial diffuse necrosis (>20%)	3

**Table 2:** Expression level of vWF, sEPCR, NO, and TNA-*α*.

	Control group		SAP group	
	12 h	24 h	12 h	24 h
vWF	0.531 ± 0.22	0.544 ± 0.2603	1.12 ± 0.17	1.2475 ± 0.13
*P*			<0.05	<0.05
sEPCR	2.254 ± 0.175	2.2477 ± 0.175	3.749 ± 0.27	4.132 ± 0.140
*P*			<0.05	<0.05
NO	5.121 ± 1.562	5.105 ± 0.856	2.729 ± 0.613	2.258 ± 0.144
*P*			<0.05	<0.05
TNF-*α*	25.28 ± 1.420	24.031 ± 10.42	70.452 ± 2.927	93.260 ± 18.431
*P*			<0.01	<0.01
Pathological store	3.67 ± 0.817	3.67 ± 0.817	6.77 ± 1.37	8.53 ± 1.26
*P*			<0.05	<0.05
Al	5.87 ± 0.15	11.04 ± 0.56	51.47 ± 23.56	68.03 ± 12.51
*P*			<0.01	<0.01
